# Retrospective Evaluation of the Effect of Lumbar Sympathetic Blockade on Pain Scores, Fontaine Classification, and Collateral Perfusion Status in Patients with Lower Extremity Peripheral Arterial Disease

**DOI:** 10.3390/medicina60050682

**Published:** 2024-04-23

**Authors:** Celalet Keser-Pehlivan, Cagatay Kucukbingoz, Umur Anil Pehlivan, Huseyin Tugsan Balli, Hakki Unlugenc, Hayri Tevfik Ozbek

**Affiliations:** 1 Department of Anesthesiology and Reanimation, Yuregir State Hospital, Adana 01240, Turkey; 2 Department of Algology, Adana City Training and Research Hospital, Adana 01370, Turkey; ckbingoz.md@gmail.com; 3 Department of Radiology, Adana Dr. Turgut Noyan Application and Research Center, Faculty of Medicine, Baskent University, Adana 01240, Turkey; uapehlivan@gmail.com; 4 Department of Radiology, Faculty of Medicine, Cukurova University, Adana 01330, Turkey; tugsanballi@gmail.com; 5 Department of Anesthesiology and Reanimation, Faculty of Medicine, Cukurova University, Adana 01330, Turkey; hunlugenc@gmail.com; 6 Department of Anesthesiology and Reanimation, Division of Algology, Faculty of Medicine, Cukurova University, Adana 01330, Turkey; hayriozbek@hotmail.com

**Keywords:** lumbar sympathetic block, Fontaine Classification, Numeric Rating Scale (NRS), Pain Detect Questionnaire (PDQ), Doppler US

## Abstract

*Background and Objectives*: The aim of this retrospective study was to evaluate the effect of lumbar sympathetic block (LSB) on pain scores, Fontaine Classification, and collateral perfusion status in patients with lower extremity peripheral artery disease (PAD), in whom revascularization is impossible. *Material and Methods*: Medical records of 21 patients with PAD who underwent LSB with a combination of local anesthetics, steroids, and patient follow-up forms containing six-month follow-ups between January 2020 and March 2021 were retrospectively reviewed. Numeric Rating Scale (NRS), Pain Detect Questionnaire (PDQ) scores, Fontaine Classification Stages, and collateral perfusion status (collateral diameter and/or development of neovascularization) evaluated by arterial color Doppler Ultrasound (US) from the medical records and follow-up forms of the patients were reviewed. *Results*: NRS and PDQ scores were significantly lower, and regression of the Fontaine Classification Stages was significantly better after the procedure at the first, third, and sixth month than at the baseline values (*p* < 0.001). Only four patients (19%) had collaterals before the procedure. An increase in the collateral diameter after LSB was noted in three out of four patients. Before the procedure, 17 patients had no prominent collateral. However, in thirteen of these patients, after LSB, neovascularization was detected during the six-month follow-up period (three patients in the first month, seven patients in the third month, and thirteen patients in the sixth month). The number of patients evolving neovascularization after LSB was found to be statistically significant at the third and sixth months compared to the initial examination (*p* < 0.001). *Conclusions*: LSB with the use of local anesthetic and steroids in patients with lower extremity PAD not only led to lower NRS and PDQ scores, but also resulted in regressed Fontaine Classification Stages and better collateral perfusion status.

## 1. Introduction

Peripheral arterial disease (PAD) is a narrowing of the arterial lumen that usually develops in the background of atherosclerosis. The most common cause of morbidity and mortality due to atherosclerosis, following coronary artery disease and stroke, is PAD. Peripheral arterial disease is an umbrella definition that includes the aortoiliac, carotid, renal, and lower extremity arterial systems that are affected. The arteries of the lower extremities are one of the most commonly affected areas in patients with PAD [1].

The prevalence and incidence of PAD increases with age [1,2,3,4]. The most important and common symptom of the patients with PAD is chronic ischemic pain caused by insufficient tissue oxygenation. The cause of insufficient tissue oxygenation is vasoconstriction in the proximal of the atherosclerotic vessels as a result of sympathetic overactivity [5,6]. The pain initially appears as walking-related, and relief is felt at rest. However, if the perfusion of the limb worsens, critical ischemic pain which is severe and persistent at rest appears [2]. Ulceration and gangrene can also accompany chronic ischemic pain. Since the patients with PAD present different symptoms and findings, they are evaluated and staged with Fontaine or Rutherford Classifications, which are usually clinical-based classifications [7]. In addition, there is also destruction and loss of sensory nerves in the skin due to arterial ulceration and tissue loss. Thus, neuropathic pain, assessed using single and/or multiple-item pain scales, can be combined with the symptoms described above [8].

Along with chronic pain in PAD, patients experience sleep and behavioral disorders (anxiety, depression), a decrease in walking distance, changes in feeding habits, and an increase in frequency of deterioration in social relations. The most effective treatment of patients with chronic limb-threatening ischemia is to establish surgical revascularization, providing vascular bypass, endarterectomy, and/or endovascular interventions [9]. In cases where revascularization is not possible, different treatment modalities are proposed to relieve pain and treat patients.

In patients with PAD in whom revascularization is impossible, the percutaneous lumbar sympathetic block (LSB) is proposed to be one of the reliable and minimally invasive treatment methods for the treatment of ischemic pain [10,11,12]. This can be performed with chemical neurolytic or local anesthetic agents, or radiofrequency ablation (RFA) [6,13]. With lumbar sympathetic ganglia block, vasodilation of arteries of the lower extremities is achieved by blocking sympathetic nerves’ overactivity and thereby increasing tissue oxygenation. Furthermore, LSB can improve tissue perfusion by increasing the collateral blood flow, and thereby the control of pain [6].

Although several studies have evaluated the effect of LSB on pain in patients with different pathologies, to our knowledge, very limited studies and data are present in the literature evaluating the effect of LSB in patients with lower extremity PAD.

The primary aim of this retrospective study was to evaluate the effect of LSB on pain (NRS and PDQ) scores in patients with lower extremity PAD in whom revascularization is impossible. The secondary aim was also to evaluate the Stages of Fontaine Classification and collateral perfusion status before and after LSB.

## 2. Materials and Methods

### 2.1. Ethics, Consent, and Permissions

This study was planned as a single-center retrospective study and was conducted in accordance with the principles of the Helsinki Declaration after the approval of the local Clinical Research Ethics Committee (decision No: 114, date: 10 September 2021). Informed consent was obtained from all patients before interventions.

### 2.2. Patient Selection

Medical records of 21 patients with PAD who underwent LSB with a combination of local anesthetics and steroids, and patient follow-up forms containing six-month follow-ups between January 2020 and March 2021, were retrospectively reviewed.

The criteria for inclusion of the patients in this study were as follows:

1. To have a diagnosis of PAD in lower extremities that could not be treated with conservative treatment (painkillers or vasodilator drugs and regular walking exercise) or revascularization procedures,
2. To have received an LSB procedure with a combination of local anesthetics and steroids,
3. To be a Stage III and/or IV according to the Fontaine Classification,
4. To have been evaluated by Fontaine Classification, the Numeric Rating Scale (NRS), and the Pain Detect Questionnaire (PDQ) score before the procedure and at the first, third, and sixth month after the procedure,
5. To have the treated lower extremities examined with arterial Doppler US at and before the first, third, and sixth month after the procedure.

Exclusion criteria from the study included the following: Patients with a history of diabetes mellitus (autonomic neuropathy can affect pain perception), Guillain–Barré syndrome, alcohol abuse, coagulopathy, and allergy to local anesthetics were excluded. Patients with signs of significant cardiovascular diseases, morbid obesity, and/or vital organ dysfunction, and patients receiving medications known to affect pain and skin infection at the site of intervention were also excluded.

### 2.3. Assessment before the Lumbar Sympathetic Block

The patients’ age, height, weight, gender, presence of additional diseases, presence of ulceration on the lower extremities, drug use (i.e., Acetylsalicylic acid, Klopidogrel, Cilostazol), smoking history, duration, and lateralization of PAD symptoms were noted. Patients’ NRS and PDQ scores and their Fontaine Classifications were reviewed and recorded. The PDQ score was determined by a nine-item questionnaire, which included gradation of pain, pain course pattern, and radiating pain [14]. Patients evaluated NRS scores as a number between 0 and 10, according to pain intensity (0: No pain; 10: The worst pain imaginable) [15]. To ensure consistency and reliability of the measurements, NRS and PDQ evaluations were conducted at rest, after a 30 min resting period. The patients were clinically staged according to Fontaine Classification (Asymptomatic: Stage I; Mild claudication: Stage IIa; Moderate–severe claudication: Stage IIb; Ischemic rest pain: Stage III; Ulcers and gangrene: Stage IV). To determine the collateral perfusion status (collateral diameter and/or development of neovascularization) of the patients, arterial color Doppler US of the lower extremities was performed by a radiologist who was blinded to clinical status. All Doppler US examinations were performed using a Logiq S8, 9L transducer (GE Healthcare, Milwaukee, WI, USA). Radiologic findings recorded before and after the procedure were evaluated and compared.

### 2.4. Procedure of Lumbar Sympathetic Block

**Figure 1:**
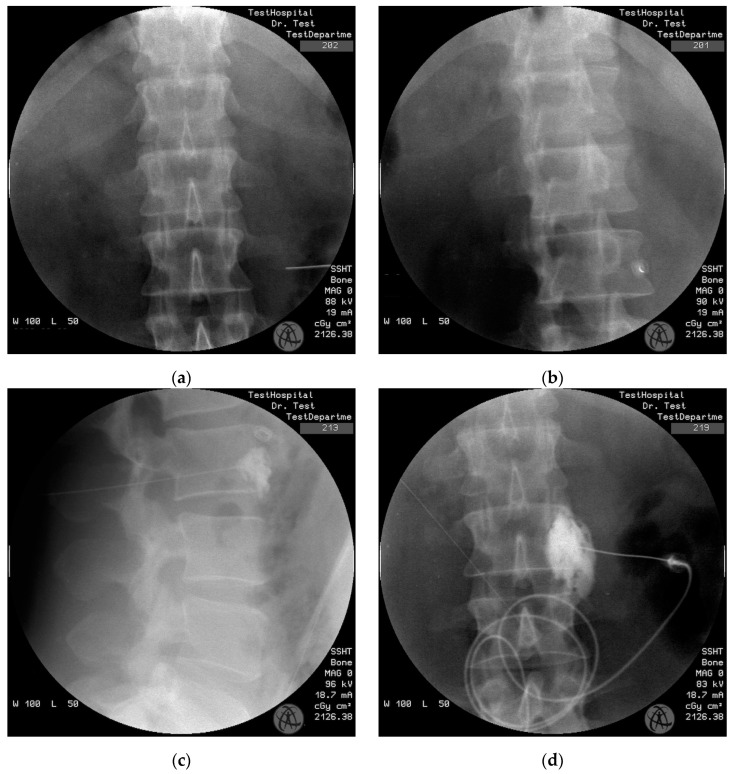
A needle was placed adjacent to the L2 vertebral body under the transverse processes in the oblique (**a**), anterior–posterior (**b**) plane under the guidance of fluoroscopy. After the administration of the contrast agent, needle placement was confirmed with lateral (**c**) and anterior–posterior (**d**) plane fluoroscopy.

Standard monitoring was applied in the operating room and an intravenous infusion of the ringer’s lactate solution was started for the patients. Patients were positioned prone, and a 15 cm high pillow was placed at the level of the lower abdomen and iliac crest to reduce lumbar lordosis. The puncture zone was cleaned according to the asepsis–antisepsis rules and closed with a sterile cover. The lower third of the second lumbar vertebra or the upper third of the third lumbar vertebra was targeted. The target lumbar vertebra was determined by anteroposterior fluoroscopic imaging, and the transverse process of the vertebra was marked. The C-arm scopy was rotated in the oblique direction until the image of the transverse process disappeared under the vertebral corpus. Then, 2 mL of 2% lidocaine subcutaneously (SC) was applied to provide local anesthesia, and a 15 cm, 21 G Chiba needle (Cook Medical Inc., Bloomington, IN, USA) was placed at the anterolateral edge of the vertebra under fluoroscopy guidance. The depth of the needle in the lateral, oblique, and anterior–posterior planes was confirmed under fluoroscopy. The possible muscular and vascular puncture was ruled out by injecting a non-ionic contrast agent (Figure 1); 10 mL of the mixture obtained from 8 mg of dexamethasone (2 mL), 80 mg of 2% lidocaine (4 mL) (Aritmal, Osel Ilac Sanayi ve Ticaret Anonim Sirketi, Istanbul, Turkey), and 20 mg of 0.5% bupivacaine (4 mL) (Buvasin, VEM Ilac Sanayi ve Ticaret Anonim Sirketi, Tekirdag, Turkey) were injected into the adjacent lumbar vertebral sympathetic ganglia under fluoroscopic guidance [16]. In the present study, all patients were already simultaneously using cilostazol to improve collateralization during LSB.

### 2.5. Follow-Up and Evaluation after Lumbar Sympathetic Block

After the procedure, patients were routinely monitored for possible complications, such as hypotension and hypoperfusion due to vasodilation and hemorrhage. Patients with no side effects or complications were discharged within six hours. Patients were advised to visit the outpatient clinics for follow-ups in the first, third, and sixth months. NRS and PDQ scores were re-evaluated to understand the effect of the procedure on the duration and intensity of pain. Patients were also reassessed with the Fontaine Classification according to their symptoms and findings. In addition, Doppler US of the lower extremity arteries was examined to assess the perfusion status of the patients during the follow-ups. Developing new collaterals (neovascularization) near the occlusive or stenotic segment compared to the initial examination (baseline) was considered a positive contribution to perfusion. All of the patients were also re-evaluated for possible complications during the follow-ups.

### 2.6. Power and Statistical Analysis

Sample size calculation was based on a power analysis. In the literature, at least a 25% decrease in the scores of NRS and PDQ after the lumbar sympathetic block is regarded as a clinically significant result [12]. Sample size needed to detect a difference in pain scores (NRS and PDQ) between baseline and after LSB at a power of 90% using a significance level of *p* < 0.05, and with an effect size of 1.35 that amounted to 13 subjects. However, all of the 21 patients who met the criteria between January 2020 and March 2021 were enrolled to compensate for losses and increase the power. The primary endpoint was defined as a 25% decrease in the scores of NRS and PDQ after the lumbar sympathetic block. The secondary aim was also to evaluate the Stages of Fontaine Classification and collateral perfusion status (collateral diameter and/or development of neovascularization) before and after the LSB.

The statistical analysis of the data was performed using the IBM SPSS Statistics Version 20.0 package program (IBM Corp. Armonk, New York, NY, USA). Categorical measurements were summarized as numbers and percentages, and numerical measurements were summarized as mean and standard deviation (median and minimum–maximum where necessary). The Mann–Whitney U test for non-normally distributed data was used to compare the numerical measurements. Generalized Estimation Equations were used to compare the change in time of measurements made at different times on the same individuals. The statistical significance level was considered as 0.05 in all tests.

## 3. Results

The demographic and clinical characteristics of the patients are summarized in Table 1.

**Figure 2:**
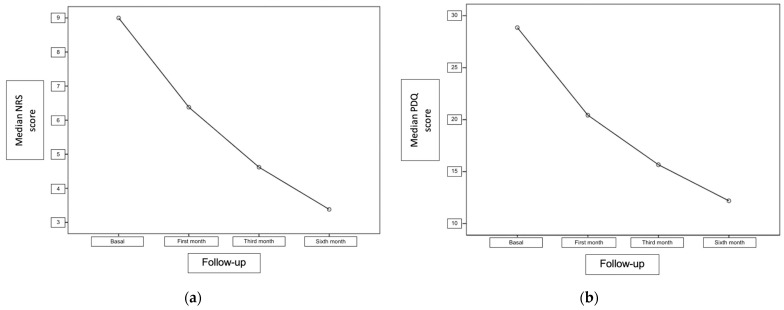
Graphical presentation of the median NRS (**a**) and PDQ (**b**) scores in follow-ups after the procedure (NRS: Numerical Rating Scale; PDQ: Pain Detect Questionnaire).

The median NRS value of the patients before the procedure was found to be 9 (min–max: 6–10). At the first, third, and sixth months of the procedure, the median NRS values dropped to 6, 5, and 3, respectively (Figure 2). The NRS values after LSB were found to be significantly lower at the first, third, and sixth months compared to baseline values (*p* < 0.001) (Table 2). Sixteen patients (76.19%) had a decrease in the NRS score of at least 50% after LSB.

The median PDQ value of the patients before the procedure was 30 (min–max: 14–37). The PDQ values decreased to 21 (min–max: 10–28), 15 (min–max: 6–28), and 11 (min–max: 2–24) at the first, third, and sixth months, respectively (Figure 2). The PDQ values after the procedure were found to be significantly lower at the first, third, and sixth month follow-ups compared to baseline values (*p* < 0.001) (Table 2). Fourteen patients (66.66%) had at least a 50% decrease in the PDQ values at the third month after LSB.

**Figure 3:**
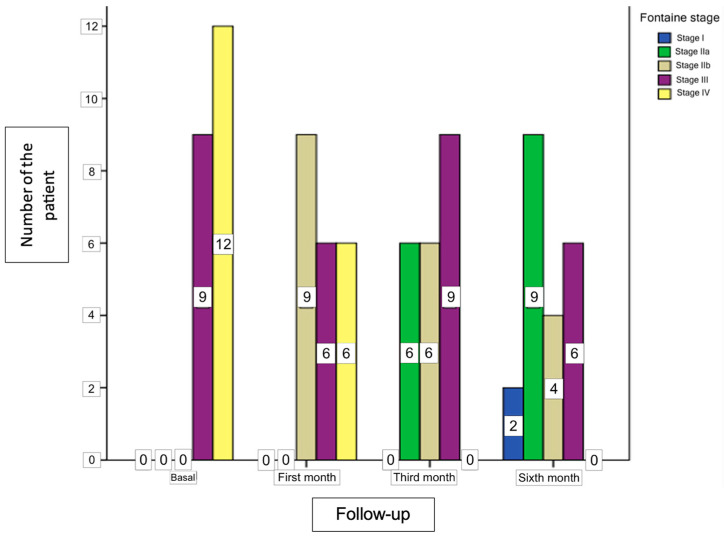
Graphical presentation of the Fontaine Stage before and after the procedure follow-ups.

The regression and Stages of Fontaine Classification of the patients before and after LSB have been demonstrated in Table 3. Before the procedure, nine patients were Stage III, whereas twelve patients were Stage IV. Regression in the Fontaine Stages after LSB was significant at the first, third, and sixth month follow-ups compared to baseline Stages (*p* < 0.001), (Table 3). Graphical presentation of the Fontaine Stages of the patients before and after the six-month follow-up period has been demonstrated in Figure 3. The number of patients exhibiting at least one Stage of regression in the Fontaine Stage after LSB was seventeen (80.95%).

**Figure 4:**
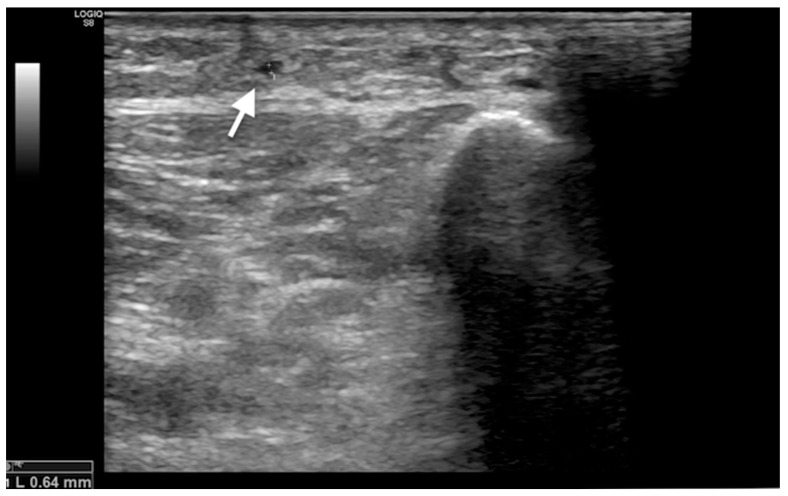
Sonographic representation of developed collateral located in the subcutaneous tissue adjacent to the stenotic segment after the lumbar symptomatic blockade procedure.

Before the procedure, only four patients (19%) had collaterals. In three out of four patients, an increase in the collateral diameter after the procedure was observed in follow-ups (one patient in the first and two patients in the third and sixth months of follow-ups). The mean diameters at baseline for the measured collaterals were 0.50 ± 0.15 mm. After LSB, the mean diameters at the first, third, and sixth months were 0.55 ± 0.1, 0.70 ± 0.1, and 0.75 ± 0.1 mm, respectively (Figure 4). One patient did not show any improvement in the collateral diameter after the procedure.

Before the procedure, 17 patients had no collateral, however, in 13 out of these patients (76.47%), after LSB, neovascularization evolved during the six-month follow-up period. After the procedure, neovascularization was detected in three out of thirteen patients (17.64%) in the first month, eleven patients (41.17%) in the third month, and thirteen patients (76.47%) in the sixth month. However, in four patients, LSB did not result in any improvement in neovascularization.

The number of patients evolving neovascularization after LSB was found to be statistically significant between baseline and the third month (*p* < 0.001), baseline and the sixth month (*p* < 0.001), the first and third months (*p* < 0.026), and the first and sixth months’ follow-ups (*p* < 0.002). However, no significant difference was found between the third and sixth months in the development of neovascularization.

## 4. Discussion

This is the first and unique retrospective study to evaluate the effect of the LSB on pain scores (NRS and PDQ), Fontaine Classification, and perfusion status in patients with lower extremity PAD in whom revascularization is impossible. The present medical records revealed that LSB with a mixture of the local anesthetics and steroids in patients with PAD not only lead to lower NRS and PDQ scores, but also resulted in regressed Fontaine Classification Stages and better collateral perfusion status.

Atherosclerosis is a dynamic process, and its prevalence increases with age and the male sex, which addresses our records that patients in our study with PAD were of advanced age, and that the gender distribution was uneven, with seventeen males and four females [1,2,3,4].

The LSB can be applied with different kinds of methods such as RFA, electrical stimulation, local anesthetics, or alcohol use for the treatment of PAD in the lower extremities [10,11,12,17,18,19,20,21,22]. We evaluated the effectiveness of LSB with a steroid and local anesthetic mixture, not only on pain scores (NRS and PDQ), but also on Fontaine Classification, collateral diameter, and development of neovascularization in patients with lower extremity PAD.

Although several studies have evaluated the effect of LSB with local anesthesia and/or a steroid combination on pain in patients with different kinds of pathologies, to our best knowledge, there is no study in the literature evaluating the effect of LSB with a steroid and local anesthetic mixture in patients with PAD in whom revascularization is impossible [23,24,25,26,27].

Bang et al. evaluated the effect of a bilateral sympathetic block with lidocaine and triamcinolone on a 12-year-old girl with primary erythromelalgia in both lower extremities whose pain was refractory to medical treatment. A bilateral sympathetic block with lidocaine and triamcinolone resulted in effective pain relief. Our experience with lower extremity PAD demonstrates that LSBs are effective in reducing pain scores and leading to regression in the Fontaine Classification Stage [23]. A systematic qualitative review conducted by Kirksey et al. described that perineural dexamethasone injection prolonged the duration of a peripheric nerve blockade with local anesthetics [24]. Spiegel et al. evaluated the effect of LSB with local anesthetic or a combination of local anesthetic and a steroid under fluoroscopy on pain relief (≥30% for at least one day) in patients with cancer-related pain in the back, abdomen, pelvis, or legs. They reported that a lumbar sympathetic blockade was effective in decreasing leg pain up to 75% [25]. Sun et al. have shown that combined treatment with continuous LSB followed by neurolysis with alcohol provided more benefits in all assessed outcomes than sympathetic alcohol neurolysis alone in the treatment of painful diabetic neuropathy [26]. Ozturk et al. presented a case report assessing LSB on pain relief in two patients with systemic drug-resistant post-herpetic neuralgia in the lower limb. In that report, both patients had at least a 50% reduction in NRS scores at the end of six months [27]. In the present study, we described an effective (≥50%) pain relief after LSB with a mixture of the local anesthetic and steroid, and our results are consistent with the literature evaluating the effect of LSB on the duration and intensity of pain in patients with PAD.

Adaptive neovascularization after arterial occlusion is an important compensatory mechanism in PAD, and includes both remodeling of pre-existing vessels to collateral vessels (angiogenesis) and angiogenic capillary growth [28]. In that situation, development of new collaterals and an increase in diameter of collaterals which results in augmented perfusion in the distal of obstruction/occlusion arteries is an important compensatory mechanism. In the present study, neovascularization in 13 of the 17 patients (76.47%) was detected at the six-month follow-up period. However, patients may show different genetic susceptibility to local cytokines and growth factors. This might be a reason why LSB did not result in neovascularization for four patients with Fontaine Classification Stage III.

Different measurement methods have been proposed to evaluate the effect of LSB on perfusion levels such as change in photoplethysmography signals or an increase in the temperature of the lower extremities [29,30]. An increase in skin temperature is frequently used to confirm the effectiveness of LSB in clinical settings. However, the assessment of skin temperature in patients with PAD occasionally may not be reliable. Kanao-Kanda et al. described improvement in perfusion with laser speckle flowgraphy (LSFG) in a patient where changes in skin temperature alone could not determine the procedure’s outcome. So, they reported that LSFG can be used to assess blood flow changes in a foot with PAD, following an LSB [31]. Contrary to photoplethysmography signals, radiological evaluation of collateralization in patients with lower extremity PAD has not been performed previously. Although three patients (17.64%) at the first month exhibited a neovascularization, the diagnostic value of Doppler US on the collateral circulation and neovascularization was clearer in the third month follow-ups than in the first month follow-ups. Although the diagnostic value of the LSB was more prominent at the sixth month of follow-up, there was no statistically significant difference in neovascularization ratios between the third and sixth months of follow-ups. Therefore, we suggest that at least three months should be waited in the case that imaging methods are used to evaluate the effect of the drugs used in the treatment modalities.

In this study, before the procedure, only four patients had collateral. The evaluation with Doppler US, which is an operator-based modality, causes subjectivity. Therefore, quantitative flow assessment might be more objective compared to diameter measurements for the assessment of collateral status. However, due to the limited sample size of patients with collateral before the treatment, we could not provide statistical data. However, studies with a larger sample size can illuminate this issue.

Some drugs used in PAD, such as Cilostazol, may show positive effects on collateralization [32]. Since all patients in the present study had used Cilostazol simultaneously during the LSB, the improvement in the collateral vessels was not attributed to solely LSB.

All data of the patients were also evaluated for possible complications of LSB, such as retroperitoneal and psoas hematoma, genitofemoral neuralgia and injury, ureteral and renal injury, and non-target injection that may occur during follow-ups [33]. No LSB-related complications at follow-up were noted during and after the procedure.

## 5. Conclusions

In our study, LSB in the treatment of pain in patients with lower extremity PAD, in whom revascularization was impossible, was an effective and safe method. The LSB with a combination of local anesthetic and steroid not only lead to lower NRS and PDQ scores, but also resulted in regressed Fontaine Classification Stages and better collateral perfusion status. A large number of further studies are needed to generalize the results of this study.

## Figures and Tables

**Table 1:** Demographic and clinical characteristics of patients.

Parameters	*n* = 21
Age ^a^	60.2 ± 11.2 61.0 (41.0–86.0)
Gender ^b^	
Male	17 (81.0)
Female	4 (19.0)
Height (cm) ^a^	170.4 ± 8.1 170.0 (156.0–185.0)
Weight (kg) ^a^	79.0 ± 12.2 81.0 (57.0–98.0)
Body mass index ^a^	27.1 ± 3.5 27.1 (19.7–32.8)
Symptomatic period (year) ^a^	4.7 ± 5.1 2.0 (1.0–20.0)

^a^ Mean ± standard deviation, median (minimum–maximum); ^b^ *n* (%).

**Table 2:** NRS and PDQ scores of patients before and after the procedure.

	Before the Procedure	First Month	Third Month	Sixth Month	*p* Value
NRS	9.0 ± 1.1 9.0 (6.0–10)	6.3 ± 1.3 6.0 (4.0–9.0)	4.6 ± 1.5 5.0(2.0–7.0)	3.3 ± 1.9 3.0 (0.0–7.0)	<0.001
PDQ	28.8 ± 6.1 30 (14–37)	20.4 ± 5.1 21 (10–28)	15.6 ± 5.3 15 (6–28)	12.1 ± 4.8 11 (2–24)	<0.001

Data were presented as mean ± standard deviation, median (minimum–maximum); NRS: Numerical Rating Scale; PDQ: Pain Detect Questionnaire.

**Table 3:** Fontaine Stages of the patients before and after the procedure.

	Before the Procedure	First Month	Third Month	Sixth Month	*p* Value
Fontaine Stages					<0.001
Stage I	-	-	-	2 (9.5%)	
Stage IIa	-	-	6 (28.6%)	9 (42.9%)	
Stage IIb	-	9 (42.9%)	6 (28.6%)	4 (19.0%)	
Stage III	9 (42.9%)	6 (28.6%)	9 (42.9%)	6 (28.6%)	
Stage IV	12 (57.1%)	6 (28.6%)	-	-	

Data were presented as *n* (%).

## Data Availability

The present data belong to and are stored at the Cukurova University Faculty of Medicine and cannot be shared without permission.

## References

[1] Criqui M.H., Matsushita K., Aboyans V., Hess C.N., Hicks C.W., Kwan T.W., McDermott M.M., Misra S., Ujueta F., on behalf of the American Heart Association Council on Epidemiology and Prevention (2021). Lower Extremity Peripheral Artery Disease: Contemporary Epidemiology, Management Gaps, and Future Directions: A Scientific Statement from the American Heart Association. Circulation.

[2] Signorelli S.S., Vanella L., Abraham N.G., Scuto S., Marino E., Rocic P. (2020). Pathophysiology of chronic peripheral ischemia: New perspectives. Ther. Adv. Chronic Dis..

[3] Shu J., Santulli G. (2018). Update on peripheral artery disease: Epidemiology and evidence-based facts. Atherosclerosis.

[4] Gardner A.W., Afaq A. (2008). Management of lower extremity peripheral arterial disease. J. Cardiopulm. Rehabil. Prev..

[5] Muller M.D., Reed A.B., Leuenberger U.A., Sinoway L.I. (2013). Physiology in medicine: Peripheral arterial disease. J. Appl. Physiol..

[6] Kulkarni K., Kulkarni R. (2021). Chemical Neurolysis and Radiofrequency Ablation of Lumbar Sympathetic Ganglion in Peripheral Vascular Diseases of the Lower Limbs. Novel Approaches in Regional Anesthesia & Pain Management.

[7] Cerqueira L.O., Duarte E.G., Barros A.L.S., Cerqueira J.R., de Araújo W.J.B. (2020). WIfI classification: The Society for Vascular Surgery lower extremity threatened limb classification system, a literature review. J. Vasc. Bras..

[8] Cruccu G., Sommer C., Anand P., Attal N., Baron R., Garcia-Larrea L., Haanpaa M., Jensen T.S., Serra J., Treede R. (2010). EFNS guidelines on neuropathic pain assessment: Revised 2009. Eur. J. Neurol..

[9] Aboyans V., Ricco J.B., Bartelink M.E.L., Martin B., Marianne B., Tina C., Jean-Philippe C., Martin C., Marco D.C., Sebastian D. (2018). 2017 ESC Guidelines on the Diagnosis and Treatment of Peripheral Arterial Diseases, in collaboration with the European Society for Vascular Surgery (ESVS): Document covering atherosclerotic disease of extracranial carotid and vertebral, mesenteric, renal, upper and lower extremity arteries Endorsed by: The European Stroke Organization (ESO) The Task Force for the Diagnosis and Treatment of Peripheral Arterial Diseases of the European Society of Cardiology (ESC) and of the European Society for Vascular Surgery (ESVS). Eur. Heart J..

[10] Joo E.Y., Kong Y.G., Lee J., Cho H.S., Kim S.H., Suh J.H. (2017). Change in pulse transit time in the lower extremity after lumbar sympathetic ganglion block: An early indicator of successful block. J. Int. Med. Res..

[11] Park S.Y., Nahm F.S., Kim Y.C., Lee S.C., Sim S.E., Lee S.J. (2010). The cut-off rate of skin temperature changes to confirm successful lumbar sympathetic block. J. Int. Med. Res..

[12] Day M. (2008). Sympathetic blocks: The evidence. Pain. Pract..

[13] Cahana A., Van Zundert J., Macrea L., van Kleef M., Sluijter M. (2006). Pulsed radiofrequency: Current clinical and biological literature available. Pain Med..

[14] Freynhagen R., Baron R., Gockel U., Tölle T.R. (2006). painDETECT: A new screening questionnaire to identify neuropathic components in patients with back pain. Curr. Med. Res. Opin..

[15] Ferreira-Valente M.A., Pais-Ribeiro J.L., Jensen M.P. (2011). Validity of four pain intensity rating scales. Pain.

[16] Gunduz O.H., Kenis-Coskun O. (2017). Ganglion blocks as a treatment of pain: Current perspectives. J. Pain Res..

[17] Punj J., Marada S. (2020). Ultrasound lumbar sympathetic block: Out of plane approach with insulated stimulation needle-Case series of three patients. Indian J. Anaesth..

[18] Marada S., Punj J., Dhar A., Bhoi D., Mohan V., Trikha A., Pandey R.K., Darlong V. (2021). To Assess Technical Feasibility of Ultrasound Lumbar Sympathetic Block with Electrical Stimulation Needle in Out of Plane Needle Orientation: A Prospective Interventional Study. Pain Med..

[19] Chahal A., Malla S., Sharma S., Chumber S., Madhusudhan K.S. (2021). CT-Guided Lumbar Sympathectomy as a Last Option for Chronic Limb-Threatening Ischemia of the Lower Limbs: Evaluation of Technical Factors and Long-Term Outcomes. AJR Am. J. Roentgenol..

[20] Dominkus M., Bauer R., Kepplinger B., Malikpur G. (1990). Percutaneous radio-frequency sympathetic block in peripheral circulatory disorders. Vasa Suppl..

[21] Singh R., Kulkarni R., Kulkarni K., Chavannavar K. (2021). Evaluation of The Radiofrequency Ablation of Lumbar Sympathetic Ganglia In Lower Limb Ischemic Ulcers In Indian Population: Radiofrequency Ablation and Lower Limb Ischemic Ulcers. Int. J. Med. Surg. Sci..

[22] Gleim M., Maier C., Melchert U. (1995). Lumbar neurolytic sympathetic blockades provide immediate and long-lasting improvement of painless walking distance and muscle metabolism in patients with severe peripheral vascular disease. J. Pain Symptom Manag..

[23] Bang Y.J., Yeo J.S., Kim S.O., Park Y.H. (2010). Sympathetic block for treating primary erythromelalgia. Korean J. Pain.

[24] Kirksey M.A., Haskins S.C., Cheng J., Liu S.S. (2015). Local Anesthetic Peripheral Nerve Block Adjuvants for Prolongation of Analgesia: A Systematic Qualitative Review. PLoS ONE.

[25] Spiegel M.A., Hingula L., Chen G.H., Legler A., Puttanniah V., Gulati A. (2020). The Use of L2 and L3 Lumbar Sympathetic Blockade for Cancer-Related Pain, an Experience and Recommendation in the Oncologic Population. Pain Med..

[26] Sun H., He M., Pang J., Guo X., Huo Y., Ma J. (2020). Continuous Lumbar Sympathetic Blockade Enhances the Effect of Lumbar Sympatholysis on Refractory Diabetic Neuropathy: A Randomized Controlled Trial. Diabetes Ther..

[27] Ozturk E.C., Sencan S., Gunduz O.H. (2021). Lumbar Sympathetic Block for Intractable Lower-Limb Postherpetic Neuralgia: Report of Two Cases. Pain Pract..

[28] Kalka C., Baumgartner I. (2008). Gene and stem cell therapy in peripheral arterial occlusive disease. Vasc. Med..

[29] Meier P.M., Zurakowski D., Berde C.B., Sethna N.F. (2009). Lumbar sympathetic blockade in children with complex regional pain syndromes: A double blind placebo-controlled crossover trial. Anesthesiology.

[30] Park S.Y., Baek H.J., Park K.S., Kim Y.C. (2014). Photoplethysmographic signals to predict the success of lumbar sympathetic blockade for lower extremity pain. J. Int. Med. Res..

[31] Kanao-Kanda M., Kanda H., Iida T., Kikuchi S., Azuma N. (2021). Clinical Application of Laser Speckle Flowgraphy to Assess Changes in Blood Flow to the Foot After a Lumbar Sympathetic Ganglion Block: A Case Report. J. Pain Res..

[32] Biscetti F., Pecorini G., Straface G., Arena V., Stigliano E., Rutella S., Locatelli F., Angelini F., Ghirlanda G., Flex A. (2013). Cilostazol promotes angiogenesis after peripheral ischemia through a VEGF-dependent mechanism. Int. J. Cardiol..

[33] Alexander C.E., De Jesus O., Varacallo M. (2022). Lumbar Sympathetic Block. 1 May 2022. StatPearls.

